# Antifouling Activity of Xylemin, Its Structural Analogs, and Related Polyamines

**DOI:** 10.1002/cbdv.202403213

**Published:** 2025-02-18

**Authors:** Hiroyoshi Takamura, Takefumi Yorisue, Kenta Tanaka, Isao Kadota

**Affiliations:** ^1^ Department of Chemistry, Graduate School of Natural Science and Technology Okayama University Okayama Japan; ^2^ Institute of Natural and Environmental Sciences University of Hyogo Sanda Japan; ^3^ Division of Nature and Environmental Management Museum of Nature and Human Activities Sanda Japan; ^4^ Research Institute for Interdisciplinary Science Okayama University Okayama Japan

**Keywords:** Amines, Antifouling activity, Barnacle, Structure–activity relationships, Xylemin

## Abstract

Biofouling, which is the accumulation of organisms on undersea structures, poses significant global, social, and economic issues. Although organotin compounds were effective antifoulants since the 1960s, they were banned in 2008 due to their toxicity to marine life. Although tin‐free alternatives have been developed, they also raise environmental concerns. This underscores the need for effective, nontoxic antifouling agents. We previously synthesized *N*‐(4‐aminobutyl)propylamine (xylemin) and its structural analogs. In this study, we assayed the antifouling activity and toxicity of xylemin, its structural analogs, and related polyamines toward cypris larvae of the barnacle *Amphibalanus amphitrite*. Xylemin and its Boc‐protected analog exhibited antifouling activities with 50% effective concentrations (EC_50_) of 4.25 and 6.11 µg/mL, respectively. Four xylemin analogs did not show a settlement‐inhibitory effect at a concentration of 50 µg/mL. Putrescine, spermidine, spermine, and thermospermine, which are xylemin‐related polyamines, did not display antifoulant effects (EC_50_ > 50 µg/mL). All evaluated compounds were nontoxic at a concentration of 50 µg/mL. These findings indicate that the size and structure of the *N*‐alkyl group are essential for the antifouling activity of xylemin. Therefore, xylemin and its analogs hold promise as nontoxic, eco‐friendly antifouling agents, offering a sustainable solution to biofouling in marine environments.

## Graphical abstract

Xylemin and Boc‐protected xylemin exhibited antifouling activities without toxicity.

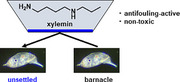

## 1 Introduction

Biofouling is a naturally occurring phenomenon caused by the settlement and accumulation of marine organisms, such as barnacles, bryozoans, and mussels, on the surfaces of undersea structures [1, 2, 3]. Biofouling negatively impacts marine industries and maritime activities worldwide, and its influences can be divided into three categories: economic, environmental and ecological, and safety [4]. The attachment and growth of fouling organisms on ship hulls cause an increase in the roughness of the hull surface, greater frictional resistance to seawater, and reduced speed and maneuverability, which results in increased fuel consumption and greenhouse gas emissions [1, 4]. In addition, the biofouling of aquaculture fishing nets deforms the nets due to the increased weight of the attached organisms and impedes the exchange of seawater and nutrients due to occlusion, thereby reducing fishery production [5]. Fouling organisms migrate with ships to seas far from their original habitats, introducing non‐native species and affecting local ecological systems [6]. Furthermore, in coastal power plants, fouling organisms block seawater pipelines, thereby decreasing the efficiency of heat exchange and cooling water systems [7]. When offshore marine oil platforms are settled by fouling organisms, they may be damaged by biological corrosion and are more susceptible to natural disasters, such as tsunamis and typhoons [8].

Various chemical and physical methods have been explored to address biofouling, including antifouling coatings [1, 4, 8, 9], electrochemical techniques [10], freshwater flushing [11], laser cleaning [12], photocatalytic methods [13], ultraviolet irradiation [14], and ultrasonic treatment [15]. Among these biofouling control techniques, antifouling coatings are the most widely used owing to their effectiveness, convenience, and low cost. Biocides have traditionally been used in antifouling coatings to eliminate fouling organisms. Organotin compounds, first introduced as biocidal components in antifouling paints in the early 1960s, became the most widely used antifouling agents [16]. However, they are nonselective and highly toxic, posing significant harm to marine ecosystems [16, 17]. In response to the growing environmental impact of organotins, the International Maritime Organization banned their use in antifouling paints on ships in 2008. Cu and Zn have been used as alternatives to organotin compounds as antifouling agents. However, metal‐based antifouling agents are toxic to several marine organisms and accumulate in marine environments [18, 19]. For example, zinc pyrithione demonstrates high toxicity in phytoplankton communities at concentrations ranging from 0.7 to 19 µg/L. Organic compounds such as Dichlofluanid, Diuron, and Sea‐Nine 211 have been used in combination with copper as metal‐free antifouling agents. Recent research has shown that organic antifoulants are toxic, indicating their negative effects on marine ecosystems [20]. For instance, Diuron exhibits toxicity against zooplankton *Artenia* larvae with an LC_50_ (50% lethal concentration) value of 30.6 mg/L. Therefore, the development of effective and environment‐friendly, nontoxic antifouling agents has received significant attention [3, 8].

**FIGURE 1:**
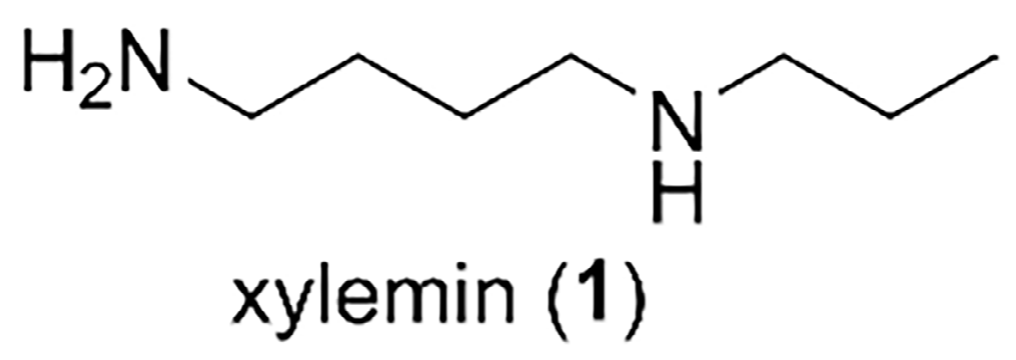
Structure of xylemin (**1**).

Natural products are regarded as promising lead compounds for eco‐friendly antifouling agents [3, 21]. Sessile marine organisms produce chemical defense substances to keep their body surfaces free of fouling. A wide variety of marine algae and invertebrates have been explored to identify novel antifouling agents with biocompatibility and nontoxic characteristics. Polyamines are aliphatic hydrocarbons with two or more amino groups [22, 23, 24]. Diamine putrescine, triamine spermidine, and tetraamine spermine are present in all living cells, from bacteria to mammals. They are involved in various biological processes, including transcription, RNA modification, protein synthesis, and the modulation of enzyme activity. We previously synthesized *N*‐(4‐aminobutyl)propylamine, named xylemin (**1**, Figure 1), and its structural analogs [25]. Biological evaluation of the synthetic compounds revealed that **1** and its analog *N*‐(4‐aminobutyl)cyclopentylamine promoted the expression of the thermospermine synthase *ACAULIS5* (*ACL5*) and enhanced xylem formation. The presence of polyamines in living cells indicates the possibility and utility of using them as nontoxic and biocompatible substances. In addition, in terms of chemical synthesis, we can synthesize polyamine **1** and its structural analogs in a short step [25]. Therefore, we decided to investigate the usefulness of **1** and its related compounds as biologically active molecules. In our continued efforts to develop environmentally friendly antifouling compounds [26, 27], we became interested in the antifouling activities of **1** and its analogs [28]. Herein, we report the antifouling activity and toxicity of **1**, its structural analogs, and related polyamines toward cypris larvae of the barnacle *Amphibalanus amphitrite*.

## 2 Results and Discussion

**FIGURE 2:**
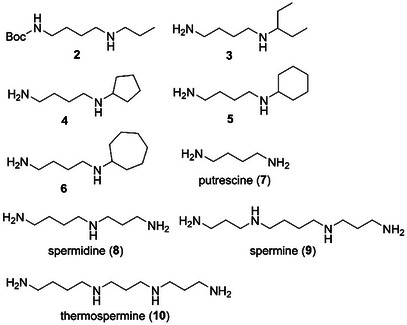
Structures of xylemin analogs **2**–**6** and xylemin‐related polyamines **7**–**10**.

First, the biological activities of xylemin (**1**) and its related compounds were assayed against the cypris larvae of the barnacle *A. amphitrite*. The larvae were treated using 24‐well polystyrene plates with 6 different concentrations of the compounds at 25°C in the dark for 96 h. The antifouling activity and toxicity of each compound are shown as the 50% effective concentration (EC_50_) and 50% lethal concentration (LC_50_) values, respectively. Xylemin (**1**) exhibited an inhibitory activity against the settlement of cypris larvae with an EC_50_ value of 4.25 µg/mL (Table 1). Encouraged by this result, we evaluated the antifouling activity of xylemin analogs. *t*‐Butoxycarbonyl (Boc)‐protected xylemin **2** (Figure 2) retained the antifouling activity with an EC_50_ value of 6.11 µg/mL, suggesting that the free primary amino group was not essential for displaying the antifouling activity of **1**. To clarify the influence of the alkyl chain moiety on the biological activity, we assayed *N*‐(4‐aminobutyl)‐1‐ethylpropylamine (**3**) and determined that this compound was antifouling‐inactive at a concentration of 50 µg/mL. (EC_50_ > 50 µg/mL). Second, we evaluated the antifouling activity of *N*‐(4‐aminobutyl)cyclopentylamine (**4**), which exhibited significant activity in promoting xylem differentiation, similar to **1** [25]. However, this analog demonstrated no inhibitory activity. In addition, *N*‐(4‐aminobutyl)cyclohexylamine (**5**) and *N*‐(4‐aminobutyl)cycloheptylamine (**6**), which differ in ring size on the nitrogen atom, also lacked antifouling activity. Although the detailed reason for the influence of alkyl chains on the biological activity is unclear at present, the relatively small size of *n*‐propyl group in **1** may be preferred for exhibiting the antifouling effect. In comparison with **1**, the relatively hydrophobic nature of **3**–**6** due to the branched alkyl chains may also affect their non‐antifouling activity.

**TABLE 1:** Antifouling activity (EC_50_) and toxicity (LC_50_) of xylemin (**1**), its analogs **2**–**6**, and related polyamines **7**–**10**.^a^

Compound	EC_50_ (µg/mL)	LC_50_ (µg/mL)
**1**	4.25	>50
**2**	6.11	>50
**3**	>50	>50
**4**	>50	>50
**5**	>50	>50
**6**	>50	>50
**7**	>50	>50
**8**	>50	>50
**9**	>50	>50
**10**	>50	>50

^a^ Against the cypris larvae of the barnacle *Amphibalanus amphitrite*.

**SCHEME 1:**
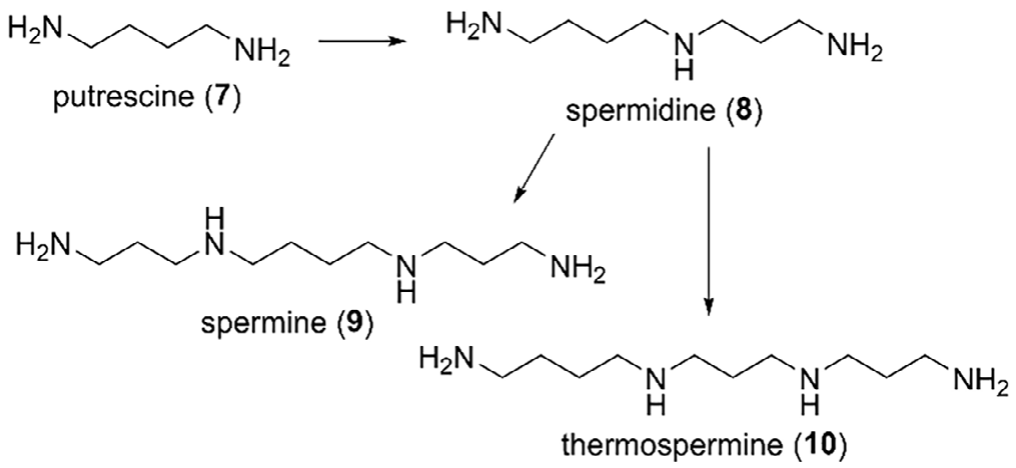
Biosynthetic pathway of polyamines.

Third, we focused on the antifouling activity of xylemin‐related polyamines. In the biosynthetic pathway, putrescine (**7**), which was synthesized from l‐ornithine or l‐arginine, was converted to spermidine (**8**) via aminopropylation by spermidine synthase (Scheme 1) [22, 23, 24]. Spermine (**9**) and thermospermine (**10**) were derivatized from **8** by the introduction of an aminopropyl group to the primary amino group on the left and right sides of **8**, respectively. Xylemin (**1**) enhanced xylem formation in various plants by competitively inhibiting biosynthesis of **10** [25, 29]. We anticipated that polyamines **7**–**10** would exhibit the same antifouling activity as compound **1**. Biological evaluation of compounds **7**–**10** revealed that all these polyamines were antifouling‐inactive. The non‐antifouling activity of **7** indicates that the *n*‐propyl substituent of **1** plays a crucial role in settlement inhibition. In addition, the non‐antifouling activity of **8**–**10** suggests that molecular size affects the antifouling activity. Pseudoceratidine, which is a natural antifouling spermidine product with two dibromopyrrole moieties, was isolated from the marine sponge *Pseudoceratina purpurea* by Fusetani et al. [28]. In the structure–antifouling activity relationship study of pseudoceratidine by Ganem et al. [30], 4,5‐dibromopyrrole‐2‐carboxylic acid exhibited antifouling activity with an EC_50_ value of 15 µg/mL toward the barnacle *A. amphitrite*, whereas two mono(dibromopyrroloyl)spermidines were inactive. These findings indicated that the structure and number of dibromopyrrole units were key factors that affect the antifouling activity of pseudoceratidine. This was supported by the results obtained in the present study, which showed that spermidine (**8**) did not exhibit antifouling activity. Finally, we examined the toxicity of compounds **1**–**10** and confirmed that they were all nontoxic at a concentration of 50 µg/mL (LC_50 _> 50 µg/mL).

## 3 Conclusions

We previously synthesized xylemin (**1**) and its analogs **2**–**6** as thermospermine (**10**) biosynthesis inhibitors. In our search for novel and environmentally benign antifouling agents, we assessed the antifouling activity and toxicity of compounds **1**–**6**, putrescine (**7**), spermidine (**8**), spermine (**9**), and thermospermine (**10**) against cypris larvae of the barnacle *A. amphitrite*. Xylemin (**1**) and Boc‐protected xylemin **2** exhibited antifouling activities with EC_50_ values of 4.25 and 6.11 µg/mL, respectively, without toxicity (LC_50_ > 50 µg/mL). Compounds **3**–**10** were antifouling‐inactive and nontoxic. These results suggested that an *n*‐propyl substituent on the nitrogen atom was required for the antifouling activity of **1**, whereas a free primary amino group was not. The creation of antifouling paints using **1** and **2** as antifouling agents and their antifouling effects in seawater will be investigated in the future. In conclusion, this study demonstrated that xylemin and its Boc‐protected analog exhibited significant antifouling activities without toxicity, suggesting their potential as eco‐friendly alternatives to traditional antifouling agents. These findings highlight the importance of further exploring xylemin‐based compounds for sustainable marine applications.

## 4 Experimental Section

Evaluation of the biological activity and statistical analysis were conducted according to the previous work [26, 27]. Adult barnacles of *A. amphitrite* attached to stones were collected in Kobe, Japan, in 2023 and maintained in aquaria. Nauplii released from adults were cultured in filtered seawater (FSW) with penicillin/streptomycin (FUJIFILM Wako Pure Chemical Corp., Osaka, Japan) and fed *Chaetoceros calcitrans* at 25°C, under a 12 h light/12 h dark cycle. The larvae reached the cypris stage after 5–6 days. Cyprids were collected, rinsed with FSW, and stored in the dark at 5°C for 3–4 days until use. The effects of the compounds on barnacle cyprids were evaluated using 24‐well polystyrene plates (Corning Inc., Corning, NY, USA). Each compound was dissolved in MeOH, and the solution was diluted with the same solvent to concentrations of 0.01, 0.03, 0.1, 0.3, 1.0, and 5.0 µg/µL. Aliquots (10 µL) of the solutions were added to the wells of the polystyrene plates and air‐dried. Each well was charged with approximately ten cypris larvae and FSW (1.0 mL). The plates were incubated at 25°C in the dark for 96 h. The number of larvae that settled (including metamorphosed larvae), died, or did not settle was counted under a microscope. Each concentration was tested in three wells, and the experiment was performed three times with different batches of larvae. The assay was conducted using CuSO_4_ (0.01, 0.03, 0.1, 0.3, 1.0, 3.0, and 10 µg) as a positive control. An assay without any compound was used as a negative control. Settlement inhibition was calculated based on negative control settlement. Antifouling activity (EC_50_) and toxicity (LC_50_) were calculated using probit analyses. EC_50_ indicates the concentration that reduces the larval settlement to 50% of the negative control. LC_50_ indicates the concentration that causes 50% mortality. If the mortality rate was not over 50% at the highest concentration of compounds (50 µg/mL), the LC_50_ value was determined to be greater than 50 µg/mL.

## Author Contributions

Hiroyoshi Takamura conceived and directed the study. Takefumi Yorisue collected and maintained the barnacles. Hiroyoshi Takamura evaluated the biological activities. Hiroyoshi Takamura prepared the manuscript. All authors analyzed the collected data.

## Conflicts of Interest

The authors declare no conflicts of interest.

## Supporting information



Supporting Information

## Data Availability

The data that support the findings of this study are available in the Supporting Information section of this article.
