# Supplementary material for: Antifouling Activity of Xylemin, Its Structural Analogs, and Related Polyamines
Source: Chem Biodivers. 2025 Feb 18;22(4):e202403213. doi: 10.1002/cbdv.202403213 (PMC12004904; doi:10.1002/cbdv.202403213)

Table of Contents

Effects of **1–10** on Settlement and Mortality of Cypris Larvae (Figure S1) S2–S5

Figure S1. Effects of **1–10** on settlement and mortality of cypris larvae.

xylemin (**1**), control settlement = 79.6%

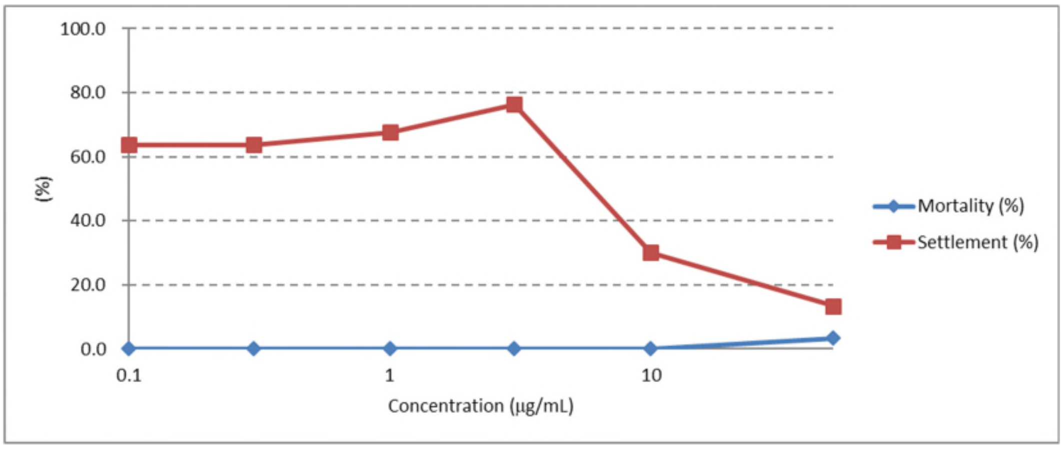

Boc-protected xylemin **2**, control settlement = 59.8%

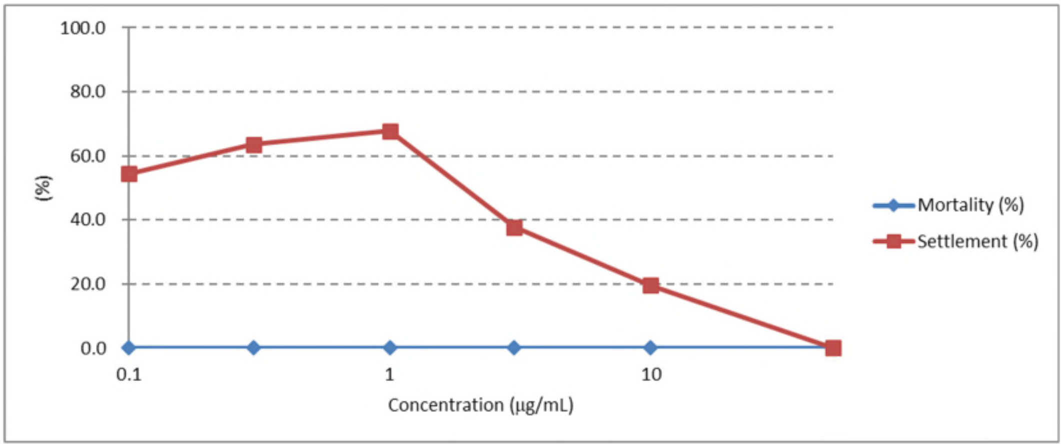

N-(4-aminobutyl)-1-ethylpropylamine (**3**), control settlement = 78.2%

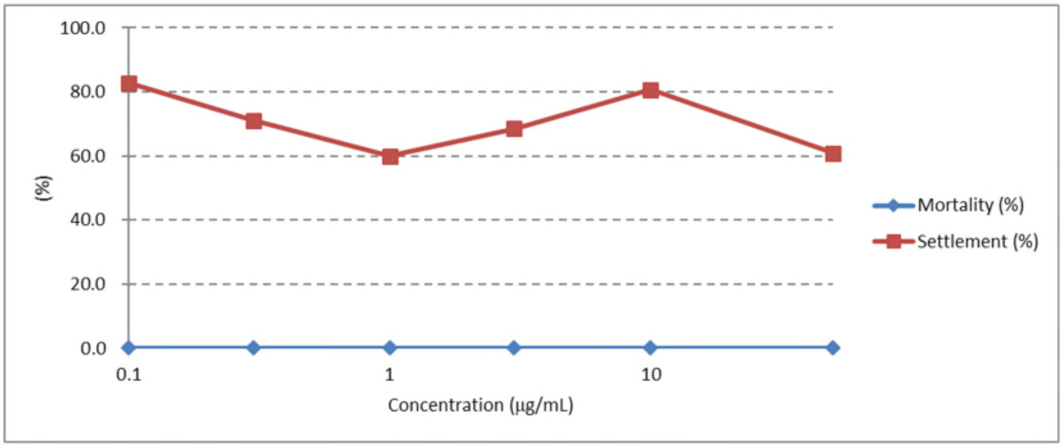

N-(4-aminobutyl)cyclopentylamine (**4**), control settlement = 59.8%

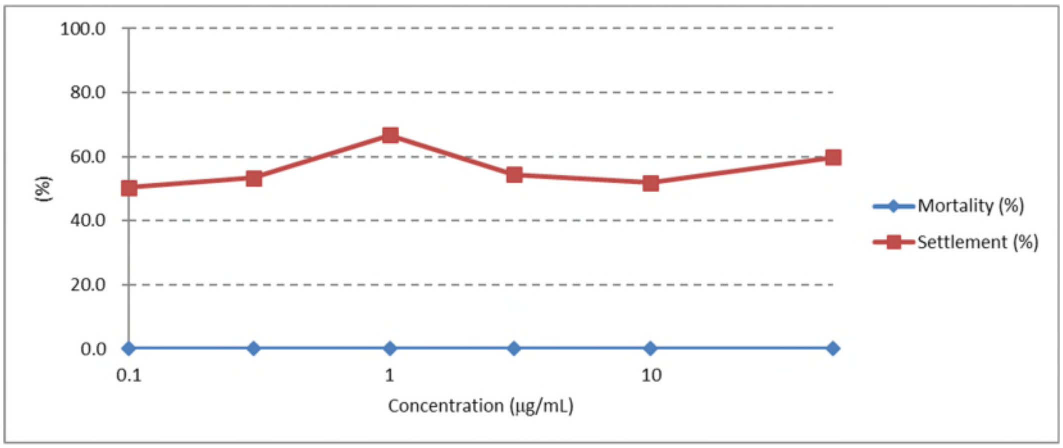

N-(4-aminobutyl)cyclohexylamine (**5**), control settlement = 79.6%

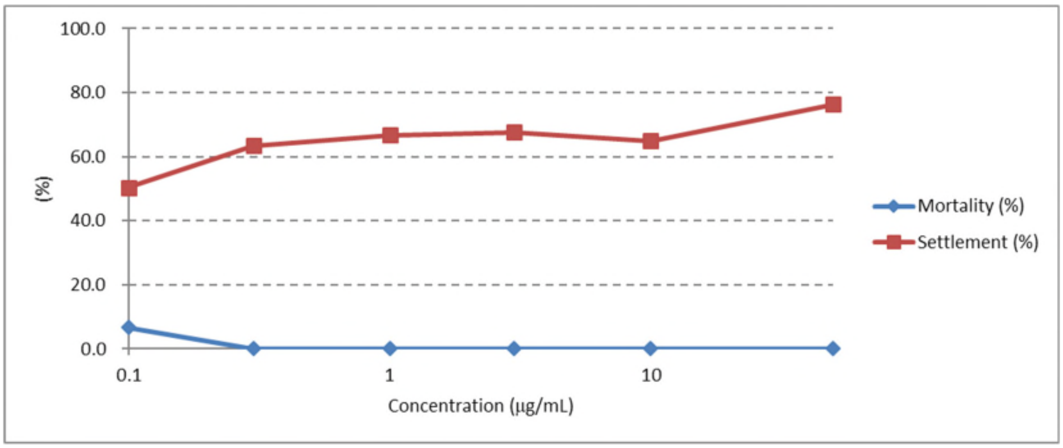

N-(4-aminobutyl)cycloheptylamine (**6**), control settlement = 59.8%

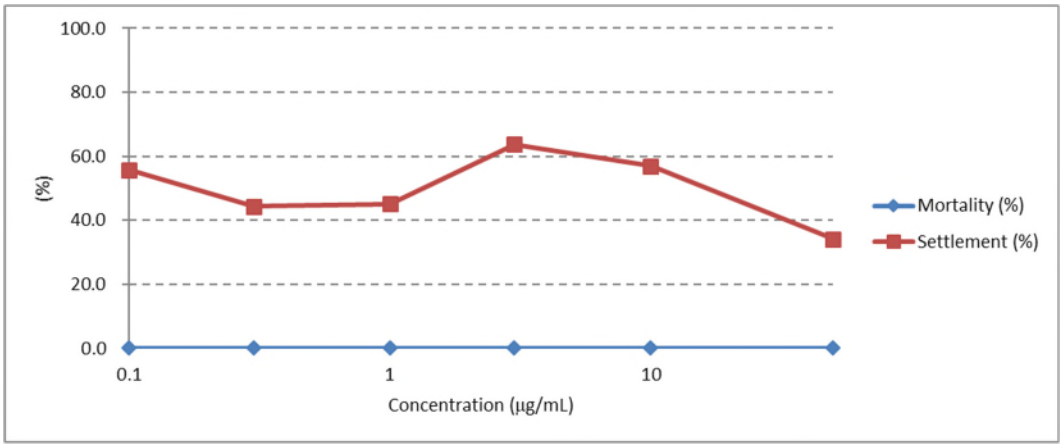

putrescine (**7**), control settlement = 77.9%

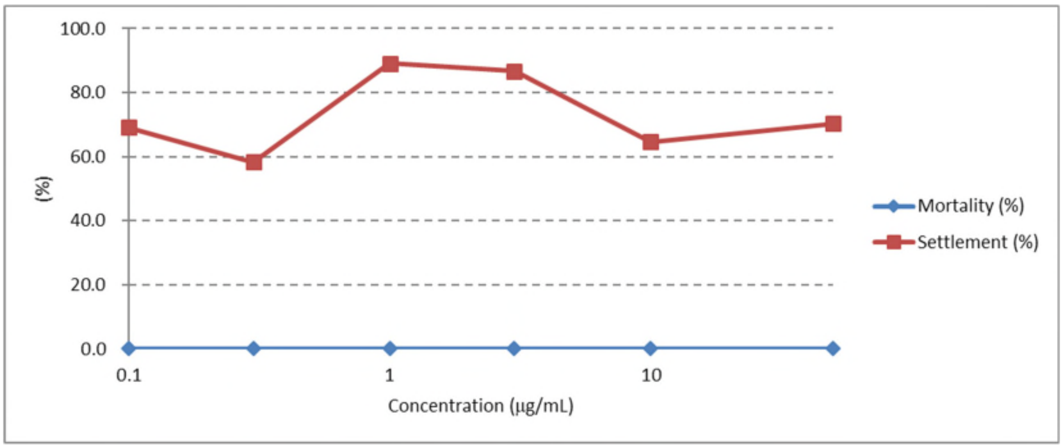

spermidine (**8**), control settlement = 77.9%

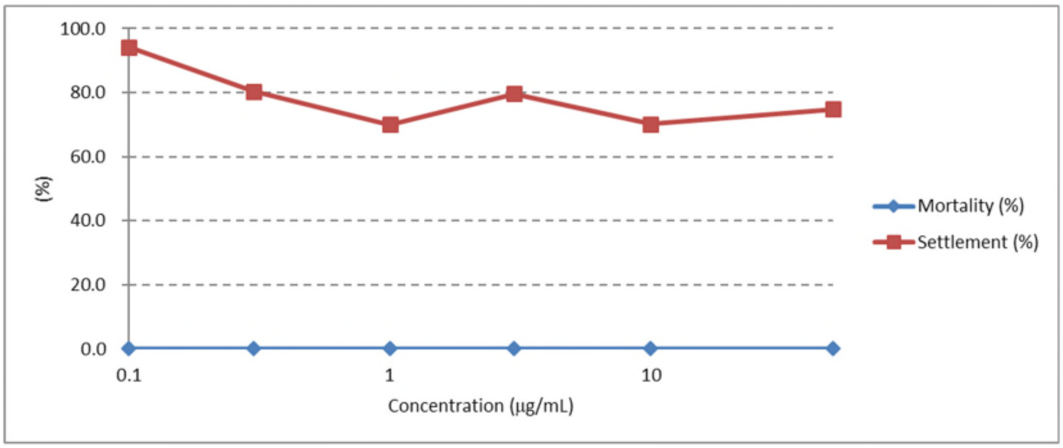

spermine (**9**), control settlement = 59.8%

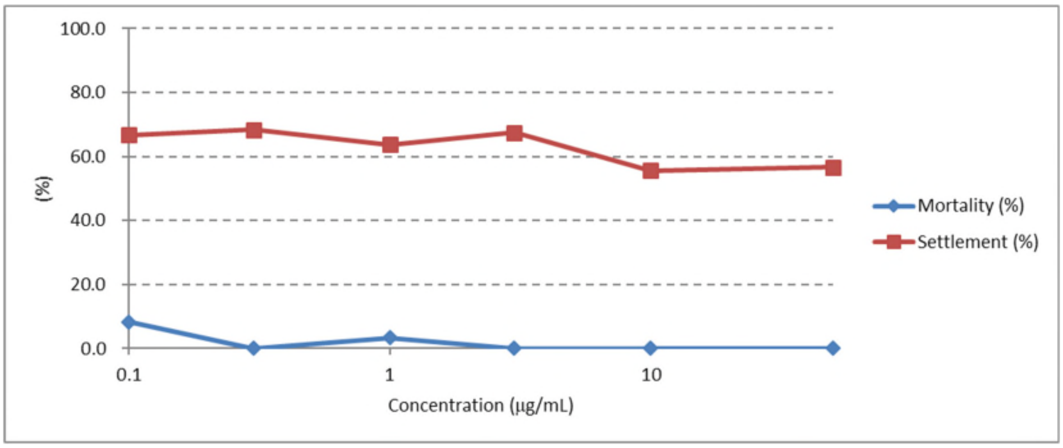

thermospermine (**10**), control settlement = 59.8%

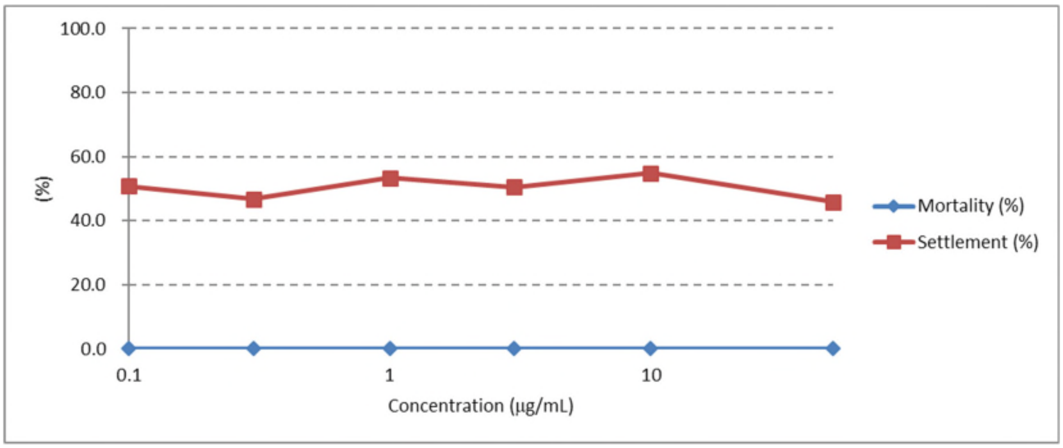

Supplement: Supplementary file 1 — Supporting Information [file CBDV-22-e202403213-s001.pdf]
